# Analyzing wealth distribution effects of artificial intelligence: A dynamic stochastic general equilibrium approach

**DOI:** 10.1016/j.heliyon.2025.e41943

**Published:** 2025-01-13

**Authors:** Fang Liu, Chen Liang

**Affiliations:** a Economic School of Changzhou University, China; b Financial Technology School of Shenzhen University, China

**Keywords:** Artificial intelligence, Wealth distribution, Heterogeneous agent dynamic general equilibrium model

## Abstract

This study explores the often-overlooked influence of artificial intelligence (AI) on wealth distribution. Using a continuous-time heterogeneous agent dynamic general equilibrium model, we investigate AI's impact on production technology as a form of biased technological progress. Our findings highlight a temporal dichotomy in AI's effects on wealth inequality: in the short term, AI exacerbates disparities in wealth distribution, while the long-term outcomes depend on the extent of AI's influence across different technological domains. The nuanced nature of AI-driven technological progress leads to distinct consequences for the rate of return on capital—showing short-term increases across all forms of technological progress, but with varying long-term effects. Based on these results, we propose policy recommendations for China to leverage AI for economic growth while mitigating inequality. This research provides a comprehensive analysis of AI's role in wealth distribution, offering valuable insights for both academic inquiry and policy formulation.

## 1 Introduction

In recent years, technological innovation, particularly in artificial intelligence (AI), has garnered global attention for its far-reaching impact on economic production. Major economies, such as China, have emphasized the importance of integrating AI with the Internet, big data, and the real economy. This policy framework positions AI as a next-generation general-purpose technology, essential for driving economic growth and aligning with national strategic priorities.

**Fig. 1:**
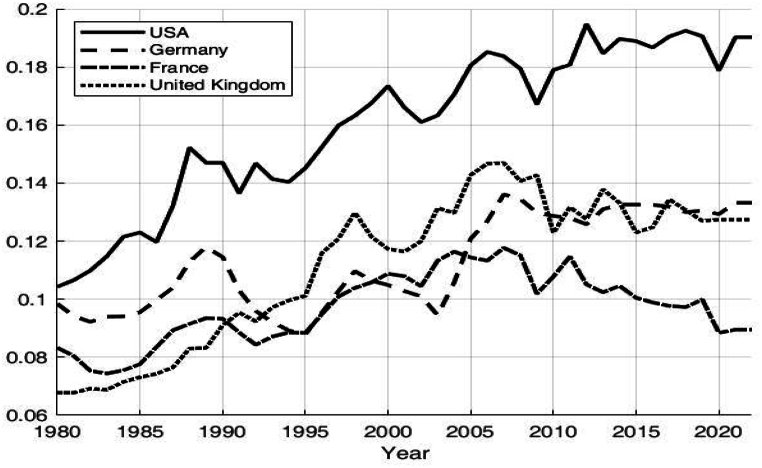
Proportion of income earned by the top 1 % of residents in western countries data source: World inequality database.

**Fig. 2:**
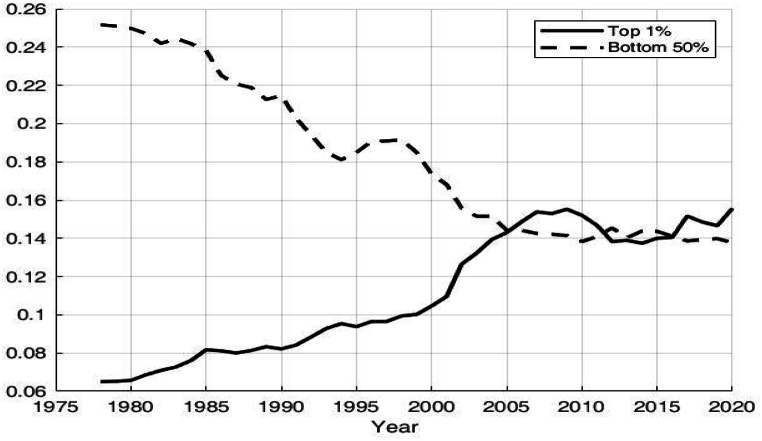
Proportion of income earned by the top 1 % and bottom 50 % of residents in China data source: World inequality database.

Historically, the global distribution of income has undergone significant changes. Income inequality began to decline after the 1950s, supporting Kuznets' hypothesis that economic growth and industrialization would reduce inequality, as illustrated in Fig. 1. However, from the 1980s onwards, this trend sharply reversed. Factors such as globalization, technological advances favoring skilled over unskilled labor, and policy changes that reduced the progressivity of the tax system have led to widening income disparities [1,2]. Similarly, China has seen rising income inequality since the reforms of 1978, as shown in Fig. 2. Rapid economic growth disproportionately benefited urban and coastal areas, exacerbating income disparities. The transition from a planned to a market economy also led to significant wage gaps, with capital accumulation concentrated in the hands of a few [3].

Despite optimism about the economic benefits of AI, scholars are increasingly concerned about its potential to worsen income inequality—a topic of renewed interest given the widening global income and wealth gaps [4,5].

Existing literature has primarily focused on the interaction between AI and labor income inequality. Some studies suggest that AI can boost labor productivity and improve wages. For example, Brynjolfsson et al. [6]argue that AI-driven automation can enhance human capabilities, thereby increasing efficiency and productivity. Similarly, Agrawal et al. [7]emphasize that AI's application across industries has the potential to increase output and value per worker, potentially raising the wages of those who can adapt to the new technology.

On the other hand, contrasting research highlights the substitution effects of AI, particularly on the low-skilled labor force. Acemoglu and Restrepo [5] argue that while AI can create new jobs, it also significantly reduces the demand for low-skilled workers, increasing their unemployment rate. Supporting this view, Autor and Salomons [8]point out that the rapid development and integration of AI technologies into production processes have disproportionately affected routine and manual labor, exacerbating income inequality and unemployment among low-skilled workers.

This divergence in viewpoints underscores the ongoing debate about AI's overall impact on the labor market. For some workers, the benefits of higher productivity and wages are offset by the risks of job displacement and higher unemployment for others. As a result, academic discourse continues as researchers seek to reconcile these opposing views and gain a more comprehensive understanding of AI's multifaceted impact on the labor market.

While current research has explored the impact of AI on labor income inequality in depth, there are notable gaps in understanding its effects on property income and wealth inequality. Existing studies tend to focus on immediate labor market outcomes, often neglecting the long-term effects of AI on wealth distribution. Addressing this gap is crucial to fully understanding the economic impact of AI.

To fill this gap, this study is guided by the following research questions: How does the integration of AI into economic production affect property income and wealth inequality in both the short and long term? Through what mechanisms does AI affect productivity and the relative returns to capital and labor in different sectors of the economy? How can policy interventions mitigate the increase in wealth inequality that may result from AI advancements?

This study makes several original contributions to address these research gaps. Firstly, it extends the analysis of AI's impact to include property income and wealth inequality, offering a more comprehensive understanding of how AI influences overall wealth distribution. Secondly, it employs a continuous-time heterogeneous agent dynamic general equilibrium model, incorporating a task-based view of production. This innovative approach combines theoretical and empirical analyses to examine the nuanced effects of AI across different economic sectors. Thirdly, the study uses Chinese data to calibrate the model, providing valuable insights into how AI affects wealth inequality in a rapidly developing economy. Lastly, it proposes actionable policy interventions to mitigate the potential exacerbation of wealth inequality due to AI advancements.

Theoretical analyses within this framework examine the nuanced effects of AI on wealth distribution by considering how AI influences the productivity of different economic sectors and the relative returns to capital and labor. Empirical analyses, including the calibration of the model using Chinese data, provide a comprehensive assessment of the initial and long-term impacts of AI on wealth inequality. The findings from these analyses offer critical policy insights for addressing economic disparities, highlighting how specific technological advancements and their integration into production processes can either exacerbate or alleviate wealth inequality. The remainder of this paper discusses the heterogeneous agent dynamic general equilibrium model, the derivation of the wealth distribution function, the model calibration using Chinese data and numerical simulations, and policy recommendations based on the findings.

## 2 Literatures

### 2.1 Artificial intelligence

Research on artificial intelligence (AI) has expanded rapidly in recent years, covering a wide range of topics from technological innovation to economic impact. Scholars have discussed in-depth research related to AI, mainly from the conceptual definition of AI. Russell and Norvig [9] provided a comprehensive definition of AI, distinguishing between weak AI, which is designed for specific tasks, and strong AI, which aims to perform any intellectual task that a human can do. This foundational work helps to clarify what constitutes AI and sets the stage for further research. In comparison, earlier definitions of AI often lacked this granularity, leading to ambiguity in both academic and practical discussions.

Another group of scholars has focused on the theoretical foundations involved in AI. Bostrom [10] explored the implications of superintelligent AI, discussing theoretical frameworks for understanding its potential risks and benefits. Tegmark [11] delved into the principles of AI development and the long-term societal impacts, proposing guidelines for ensuring that AI technologies align with human values and ethical standards. These theoretical studies build on the initial works of Turing [12] and McCarthy [13], who laid the groundwork for AI but did not fully address the broader societal implications.

A small number of scholars have identified applications and potential consequences of AI in various economic and social contexts. Acemoglu and Restrepo [5] examined the impact of AI and automation technologies on economic growth and employment, finding that while AI can significantly increase productivity, it may also exacerbate wealth inequality. This aligns with but also extends the findings of Autor et al. [14], who identified the displacement effects of technology on labor markets. Aghion et al. [15]further examined how different types of technological advances (e.g., capital-augmenting technologies and Hicks-neutral technological advances) affect economic growth and wealth distribution, concluding that capital-augmenting technologies can partially mitigate wealth inequality. This research builds on earlier studies by Brynjolfsson and McAfee [16], who emphasized the dual nature of technological impacts but did not differentiate between types of technological progress. Zhang et al. [17]analyzed the potential of AI as a catalyst for economic expansion in emerging markets, with a particular focus on China. They emphasized the need for targeted policies to harness the benefits of AI while addressing its adverse effects on inequality, adding a new dimension to the broader discussions by providing region-specific insights.

Despite these advancements, there are still gaps in the research. The conceptual definitions provided by early scholars often lack specificity, making it difficult to apply them in practical scenarios. Theoretical research on AI, while insightful, sometimes fails to bridge the gap between abstract concepts and real-world applications. For example, while Turing and McCarthy set the foundation, their work did not foresee the complex ethical and societal challenges discussed by Bostrom and Tegmark. Additionally, many studies tend to focus on either the technological or economic impacts of AI without adequately addressing how these two areas intersect and influence each other. This gap is evident when comparing the isolated technological focus of early AI research with the integrated socio-economic analyses seen in more recent studies. Future research should aim to provide more precise definitions of AI, develop theoretical frameworks that are closely linked to practical applications, and explore the interplay between technological advancements and economic outcomes comprehensively. Integrating these aspects can help bridge the gap between abstract theoretical constructs and their tangible impacts on society.

Given the observations of wealth inequality and the differential impacts of AI across sectors, we propose the following hypothesis.

Hypothesis 1 AI-driven technological progress exacerbates wealth inequality, particularly between capital income and labor income.

### 2.2 Relationship between artificial intelligence and the labor market

The impact of AI on the labor market has been a major focus of recent research, but findings in this area are often contradictory. Some scholars highlight the positive aspects of AI, emphasizing how it can create new job opportunities and increase demand for advanced skill sets. Frank et al. [18] argues that while AI can lead to job losses in certain sectors, it also opens new opportunities in others, thus having a net positive effect on employment. They suggest that the demand for new skills and the creation of new types of jobs can offset the negative impacts of automation on traditional roles.

Conversely, other researchers are more pessimistic about the labor market effects of AI. Acemoglu and Restrepo [5] contend that AI-driven automation tends to benefit high-skill labor while reducing opportunities for routine manual jobs, thereby exacerbating income inequality. Similarly, Nedelkoska and Quintini [19] estimate that a significant percentage of jobs are at risk of being automated, highlighting the potential for widespread job displacement and increased unemployment.

Further adding to the complexity, Webb [20] underscores the importance of policy interventions to manage the transition towards an AI-driven economy. He argues that without proactive measures such as education and training programs, the negative impacts of AI on the labor market could outweigh the benefits. This perspective suggests that the outcomes of AI adoption are highly contingent on the effectiveness of accompanying policies. Sachs et al. [21]provide another layer of complexity by examining the impact of AI in emerging markets. They find that while AI has the potential to drive economic growth, it also poses significant challenges related to inequality and job displacement. This regional analysis indicates that the effects of AI are not uniform across different economic contexts, further complicating the overall assessment of AI's impact on the labor market.

These conflicting perspectives highlight the inconsistencies and unresolved debates within current research. Some studies present AI as a catalyst for economic and job growth, while others emphasize the risks of increased inequality and job displacement. The divergent findings suggest that the impact of AI on the labor market is multifaceted and context-dependent, requiring a nuanced approach to policy and further empirical research to reconcile these differences and provide a clearer understanding of AI's role in shaping the future of work.

Despite the extensive research on the impact of AI on the labor market, several gaps and areas for further exploration remain. The existing body of literature presents conflicting views on whether AI is ultimately beneficial or detrimental to the labor market, highlighting the need for more nuanced and comprehensive studies. One major gap in the current research is the lack of longitudinal studies that examine the long-term effects of AI on employment and wage dynamics. Most existing studies focus on short-term impacts, leading to an incomplete understanding of how AI-driven automation will reshape the labor market over extended periods. Future research should employ longitudinal data to track the career trajectories of workers displaced by AI and the evolution of newly created job roles.

There is a need for more region-specific studies that consider the varying impacts of AI across different economic contexts. As highlighted by Sachs et al. [21], the effects of AI are not uniform and can vary significantly between developed and emerging markets. Investigating these regional differences can provide more tailored insights and help policymakers design region-specific strategies to manage AI's impact on the labor market.

Furthermore, existing studies often overlook the qualitative aspects of job changes brought about by AI. While many focus on job quantity, there is limited research on how AI might alter job characteristics, working conditions, and career trajectories. Future research should address these qualitative aspects to provide a more holistic view of AI's impact on the labor market. Lastly, empirical validation of theoretical models is essential. Many current studies are based on theoretical predictions without sufficient empirical evidence. Conducting empirical studies to validate these models will provide concrete evidence of AI's effects on employment dynamics, thus bridging the gap between theory and practice.

Based on these insights, we hypothesize.

Hypothesis 2 AI's impact on income distribution is largely driven by capital-augmenting technological progress, which increases capital income relative to labor income.

### 2.3 AI's impact on capital share and income distribution

The relationship between AI, net capital share, and income distribution has emerged as a critical area of research, with scholars exploring how AI technologies affect economic factors through compositional effects and intra-factor share changes. This section reviews the literature on how AI influences the distribution of income by altering capital and labor shares.

Some studies highlight the positive impacts of AI on capital share by emphasizing the compositional effects of technological advancement. Brynjolfsson et al. [6]argue that AI-driven automation enhances productivity and efficiency, leading to a higher capital share as businesses invest more in AI technologies. This shift results in a compositional change where capital-intensive sectors grow faster, thereby increasing the overall capital share in the economy. Their research shows that sectors heavily investing in AI tend to exhibit higher growth rates, supporting the notion that AI technology benefits capital accumulation and returns. Similarly, Gordon [22] found that AI investments can spur economic growth by enabling more efficient production processes, which enhances the returns on capital. Furthermore, Anton Korinek and Joseph E. Stiglitz [23] discussed how AI and automation can lead to a capital-biased technological change, increasing the share of income going to capital owners.

On the other hand, researchers like Acemoglu and Restrepo [5] have highlighted the intra-factor share changes caused by AI. They found that while AI can increase overall productivity, it often does so at the expense of labor's share of income. This shift occurs because AI and automation replace routine manual jobs, reducing the demand for labor in certain sectors. As a result, the capital share increases relative to the labor share, exacerbating income inequality. Their study emphasizes the importance of considering both the direct productivity effects and the distributive impacts of AI technologies on different factors of production. In a similar vein, De Loecker et al. [24] explored how market concentration driven by AI technologies can lead to higher profits for capital owners at the expense of wages. Autor et al. [25]also found that technological advancements, including AI, tend to favor capital over labor, leading to a declining labor share of income.

Complementing these views, Eden and Gaggl [26] explore how the adoption of AI technologies affects the distribution of income within capital and labor categories. They argue that AI can lead to a more pronounced concentration of capital ownership, where returns are increasingly accrued by a smaller group of high-capital investors. This concentration effect magnifies income inequality as the benefits of AI are not evenly distributed across all capital owners. Similarly, within the labor market, AI disproportionately benefits high-skill workers while reducing opportunities for low-skill workers, leading to greater wage disparity. Bessen [27] supports this by demonstrating that AI technologies create significant wage gaps between different skill levels, further contributing to income inequality. Brynjolfsson, McAfee, and Spence [16] also noted that the differential impact on various labor segments intensifies income disparities.

Further complicating the analysis, Gutiérrez and Piton [28]examine the regional variations in the impact of AI on capital share and income distribution. Their study shows that in regions with high levels of AI adoption, the capital share tends to increase significantly, contributing to rising income inequality. However, in regions with lower AI penetration, the effects are less pronounced. This finding suggests that the impact of AI on income distribution is not uniform and depends on the extent of AI integration into the local economy. This regional discrepancy is echoed by Atkinson [1], who found that the benefits of AI-driven growth are unevenly distributed, often favoring developed regions over developing ones.

These conflicting perspectives and findings highlight the complexities and unresolved debates within current research. Some studies present AI as a catalyst for economic growth and higher capital share, while others emphasize the risks of increased income inequality and reduced labor share. The divergent findings suggest that the impact of AI on capital share and income distribution is multifaceted and context-dependent, requiring a nuanced approach to policy and further empirical research to reconcile these differences.

Despite the extensive studies, several gaps and areas for future exploration remain. There is a lack of longitudinal studies that examine the long-term effects of AI on capital and labor shares, leading to an incomplete understanding of how AI-driven automation will reshape income distribution over extended periods. Future research should employ longitudinal data to track these dynamics comprehensively. Additionally, more region-specific studies are needed to understand the varying impacts of AI across different economic contexts. As highlighted by Gutiérrez and Piton [28], the effects of AI are not uniform and can vary significantly between regions. Investigating these regional differences can provide more tailored insights and help policymakers design region-specific strategies to manage AI's impact on income distribution.

Furthermore, existing studies often overlook the qualitative aspects of income distribution changes brought about by AI. While many focus on quantitative shifts in capital and labor shares, there is limited research on how AI might alter the qualitative aspects of income, such as job quality and career trajectories within the labor market. Future research should address these qualitative aspects to provide a more holistic view of AI's impact on income distribution. Lastly, empirical validation of theoretical models is essential. Many current studies are based on theoretical predictions without sufficient empirical evidence. Conducting empirical studies to validate these models will provide concrete evidence of AI's effects on income distribution, thus bridging the gap between theory and practice.

Building on this, we propose.

Hypothesis 3 The introduction and adoption of AI technologies increase regional wealth inequality, with technologically advanced regions experiencing higher capital returns and greater wealth concentration.

**Fig. 3:**
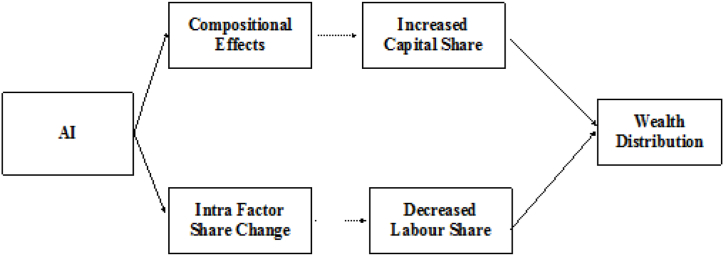
Logical framework.

Based on the literature review analysis, the logical framework diagram of this paper is shown in Fig. 3.

## 3 Model

### 3.1 Residents

This article examines the impact of artificial intelligence (AI) technology on wealth distribution within the economy, utilizing a heterogeneous individual general equilibrium model framework. Our model adopts Blanchard's perpetual youth model [29] to account for individual heterogeneity in the life and death processes of residents. We assume uniform ability among residents who earn income from both labor and capital markets throughout their lifespan. Mortality among residents is modeled as a Poisson process with a death rate P, indicating a probability P∗dt of death at any given instant.

Residents aim to maximize their lifetime utility represented by the following integral:

(1) $$\int_{0}^{\infty }e^{-\left(\vartheta +P\right)t}\frac{{C\left(t\right)}^{1-\theta }}{1-\theta }dt$$

In equation (1), let the subjective discount rate be denoted as ϑ∈(0,∞), the relative risk aversion coefficient as θ ∈(0,∞) and the death probability of residents per unit time as P∈(0,1) . The variable C(t) signifies the consumption level of residents at time t.

The budget constraint of individual residents satisfies:

(2) $$\dot{a}\left(t\right)=W\left(t\right)+r\left(t\right)a\left(t\right)-C\left(t\right)$$

In equation (2), where α represents the individual's financial wealth, $\dot{a}$ represents the rate of change of individual financial wealth, a(t) defines financial wealth of the individual at time t, $\dot{\;a}\left(t\right)$ represents rate of change of individual financial wealth at time t. W(t) presents income at time t, and r(t) defines real interest rate at time t. Residents consume all their accumulated wealth upon death without leaving any inheritance, and the financial wealth of newly born residents is zero.

### 3.2 Manufacturers

The study also explores AI technology from the production perspective. Following the framework by Aghion et al. [15], AI's impact on production encompasses automation technology, Hicks-neutral technology, capital-enhancing technology, and labor-enhancing technology. We model the total output of the economy as produced by a set of tasks with a constant elasticity of substitution:

(3) $$Y\left(t\right)=A\left({\int_{0}^{1}Z_{i}{\left(t\right)}^{\frac{\varepsilon -1}{\varepsilon }}di}^{\frac{\varepsilon }{\varepsilon -1}}\right)$$

In equation (3), Y(t) presents total output at time t. A defines technology level. The elasticity of substitution between tasks ε∈(0,∞), and the $Z_{i}\left(t\right)$ represents output of task i at time t. Each task $Z_{i}$ is either produced using capital or labor, depending on automation status and relative production costs [30]. The production for a single task $Z_{i}$ is given by:

(4) $$Z_{i}=\left\{ \begin{array}{c} A_{K}K_{i}\left(t\right)+A_{L}L_{i}\left(t\right)\text{If}\;\text{automation}\;\text{is}\;\text{achieved}\\ {\;A}_{L}L_{i}\left(t\right)\text{If}\;\text{automation}\;\text{is}\;\text{not}\;\text{achieved} \end{array}\right.$$

In equation (4), AK and AL represent capital-enhancing and labor-enhancing technologies, respectively. $K_{i}\left(t\right)$ and $L_{i}\left(t\right)$ represent capital used for task i at time t and labor used for task i at time t,respectively. To ensure that the increase in automation levels always has a positive effect on production [31], it is assumed that the cost-effectiveness ratio of automated tasks using capital $R/A_{K}$ is always less than or equal to the ratio for tasks using labor $W/A_{L}$, where R is the capital rental rate, r the real interest rate, and δ the depreciation rate. Therefore, R=r+δ.

The aggregate production function, considering automation, transforms into:

(5a) $$Y\left(t\right)=A{\left(\beta^{\frac{1}{\epsilon }}{\left(A_{K}K\left(t\right)\right)}^{\frac{\epsilon -1}{\epsilon }}+{\left(1-\beta \right)}^{\frac{1}{\epsilon }}{\left(A_{L}L\left(t\right)\right)}^{\frac{\epsilon -1}{\epsilon }}\right)}^{\frac{\epsilon }{\epsilon -1}}$$

In equation (5a), β defines proportion of tasks that have been effectively automated,^1^ and K(t) presents total capital used in production at time, L (t) presents total labor used in production at time.

## 4 General equilibrium solution

The dynamic evolution of wealth distribution under the influence of artificial intelligence (AI) is inherently complex, reflecting the multifaceted ways AI interacts with economic variables. This complexity arises from AI's dual role as both a productivity enhancer and a disruptor of traditional economic structures, necessitating a nuanced analytical approach [4,5]. Given the model's ability to derive an analytical solution for the wealth distribution function under stable conditions, the first five sections of this chapter focus on solving the model within the framework of a steady-state general equilibrium. The sixth section then explores deviations from this equilibrium, highlighting the dynamic adjustments and potential instabilities in the wealth distribution process influenced by AI.

### 4.1 Residents' optimization

The antecedent section outlined the optimization predicament of lifetime utility for individual residents within the economic context, formulated in the framework of the Hamilton-Jacobi-Bellman equation:

(5b) $$\rho V\left(a\right)=\max\frac{c^{1-\theta }}{1-\theta }+V^{\prime }\left(a\right)\left[W+ra-c\right]$$

$$st:\dot{a}=W+ra-c$$

In a state of economic equilibrium, the actual interest rate r and wage W remain constant. Solving the aforementioned Hamilton-Jacobi-Bellman (HJB) equation at this juncture provides the policy function and total wealth accumulation function for living residents [5]:

(6) $$c=\left[r-\left(\frac{r-\rho }{\theta }\right)\right]x$$

(7) $$\dot{x}=\left(\frac{r-\rho }{\theta }\right)x$$

Equation (6) delineates the consumption policy function for residents, while equation (7) articulates the accumulation equation for their total wealth, where x = W/r+a signifies the total wealth owned by an individual resident. The definition of total wealth x reveals that, for an economic individual, their total wealth can be partitioned into two components: human wealth h = W/r and financial wealth a. Over the course of a resident's lifetime, their total wealth experiences growth in accordance with the expression in equation (7). Upon a resident's demise, wealth is promptly consumed. The occurrence of death is stochastic and adheres to a Poisson process.

(8) $$W=\left({\left(1-\beta \right)}^{\frac{1}{\epsilon }}A_{L}^{\frac{\epsilon -1}{\epsilon }}\right)A^{\frac{\epsilon -1}{\epsilon }}{\left(\frac{Y}{L}\right)}^{\frac{1}{\epsilon }}$$

(9) $$R{=\beta }^{\frac{1}{\varepsilon }}{{\;A}_{K}}^{\frac{\varepsilon -1}{\varepsilon }}A^{\frac{\varepsilon -1}{\varepsilon }}{\left(\frac{Y}{K}\right)}^{\frac{1}{\varepsilon }}$$

### 4.2 Optimization of firms

Under the assumption of perfect competition in the product market, wherein all firms realize zero economic profit, capital and labor receive returns commensurate with their marginal productivity. Adopting total output as the general numeraire and normalizing its price to 1, the inverse demand functions for capital and labor within the total product firm, along with the price index for the total product, are derived from equation (5):

(10) $$1=P{=A}^{-1}{\left[\beta {{\;A}_{K}}^{\varepsilon -1}R^{1-\varepsilon }+\left(1-\beta \right){{\;A}_{L}}^{\varepsilon -1}W^{1-\varepsilon }\right]}^{\frac{1}{1-\varepsilon }}$$

When the quantities of capital (*K*) and labor (*L*) are predetermined, equations (8), (9), (10) collectively establish the equilibrium conditions for the factor markets in a state of steady equilibrium.

### 4.3 General equilibrium

The clearance of the financial market implies that the capital held by firms in the economy can be considered as the financial wealth purchased by residents. By summing up the savings decisions of all individual residents in the economy, we obtain the accumulation equation (11) for the overall wealth in the economy:

(11) $$\dot{X}=\frac{r-\rho }{\theta }X-pK$$

In equation (11), the term (r−ρ)X/θ on the right-hand side signifies the rate of wealth growth determined by optimal savings decisions of individual residents, while *-pK* denotes the overall wealth reduction resulting from resident fatalities. The demise of individual residents follows a Poisson process with a rate of *ρ*, and the wealth reduction due to individual resident deaths adheres to a compound Poisson process $S=\sum_{0}^{N\left(t\right)}\left(-a\right)$, where *S* denotes the cumulative wealth decrease due to individual resident deaths. According to the law of large numbers, the aggregate wealth reduction at any given moment in the economy due to resident deaths is the sum of the expected wealth reduction resulting from individual resident deaths, which is *−pK*.

Economic equilibrium necessitates the constancy of per capita capital, real interest rates, and wages. Given a constant population, in equilibrium, the left-hand side of equation (11) equals 0. Consequently, we arrive at equation (12):

(12) $$0=\frac{r-\rho }{\theta }\left(K+\frac{WL}{r}\right)-pK$$

Equation (12) fundamentally conveys information pertaining to the capital supply within the economic framework. By normalizing the population quantity in the economy to 1 and introducing certain adjustments, we can derive the capital supply function normalized by wages, denoted as equation (13). Analogously, the optimization conditions for producers furnish insights into the factor demand function. Through specific transformations applied to equations (8), (9), we can deduce the capital demand function normalized by wages, represented by equation (14).

(13) $${\left(\frac{K}{W}\right)}^{S}=\frac{1-\frac{\rho }{r}}{\left(\theta p+\rho -r\right)}$$

(14) $${\left(\frac{K}{W}\right)}^{D}={\left[\frac{{\left({\left(\left(r+\delta \right)/\beta^{\frac{1}{\varepsilon }}\;\left({AA}_{K}\right){}^{\frac{\varepsilon -1}{\varepsilon }}\right)}^{\varepsilon -1}-{\beta^{\frac{1}{\varepsilon }}\;\left({AA}_{K}\right)}^{\frac{\varepsilon -1}{\varepsilon }}\right)}^{\varepsilon }}{{1-\beta \;\left({AA}_{K}\right)}^{\frac{\varepsilon -1}{\varepsilon }}{\left(r+\delta \right)}^{1-\varepsilon }}\right]}^{\frac{1}{1-\varepsilon }}$$

Achieving market clearance necessitates the equating of the capital supply function to the demand function. The amalgamation of equations (13), (14) yields equation (15):

(15) $$\left(r-\rho \right){\left(r+\delta \right)}^{\varepsilon }-\delta {\beta \left({A\;A}_{K}\right)}^{\varepsilon -1}\left(r-\rho \right)+\rho \theta {\beta \left({A\;A}_{K}\right)}^{\varepsilon -1}r=0$$

Equation (15) exclusively involves the endogenous variable, the real interest rate *r*, from which the value of *r* can be ascertained, and other variables in the model can be expressed in terms of *r*. By utilizing equation (13), a relationship between the interest rate and the net capital share can be established, as expressed in equation (16):

(16) $$r=\rho +\theta p\sigma_{Knet}$$

Here, $\sigma_{Knet}$ in equation (16) represents the net capital share, specifically defined as $\sigma_{Knet}$ = *rK/(rK + WL*).

### 4.4 Wealth distribution

In the economy, there are numerous residents, and the total wealth possessed by each resident at a specific moment can be regarded as a random variable, denoted as *x*. The distribution of *x* provides all the information about wealth distribution in the economy. The rules and models governing the accumulation of individual total wealth *x* and the birth-death process specified by them allow the calculation of the corresponding Kolmogorov forward equation, resulting in the pattern of the distribution of *x* over time in the steady state, as shown in Equation (17):

(17) $$\frac{\partial g\left(x,t\right)}{\partial t}=-{\left(\frac{r-\rho }{\theta }xg\left(x,t\right)\right)}^{\prime }\;x-pg\left(x,t\right)$$

This equation describes how the distribution function of individual wealth evolves over time. The first term on the right side describes the changes in the probability density function caused by the process of individual total wealth accumulation, and the second term describes the changes in the probability density function caused by the death process of individuals. As the economy converges to a steady state, *x* (*t*) will probabilistically converge to a stationary process, resulting in ∂*g*(*x,t*)/∂*t* = 0 in the steady state. If*x* follows a Pareto distribution *g*∗(*x*) in the steady state and substituting it into Equation (17), the distribution density function of individual total wealth can be calculated as:

(18) $$\left\{ \begin{array}{c} 0,if\;x<\frac{W}{r}\\ \frac{\frac{1}{\sigma_{Knet}}{\left(\frac{W}{r}\right)}^{\frac{1}{\sigma_{Knet}}}}{x^{\frac{1}{\sigma_{Knet}}+1}},ifx\geq \frac{W}{r} \end{array}\right.$$

The probability density function of individual wealth under steady-state conditions can be further utilized to compute pertinent indicators assessing wealth distribution. This study employs two metrics to gauge wealth inequality: the top share of wealth and the Gini coefficient. Initially, we calculate the tail distribution of individual total wealth:

(19) $$\mathrm{Pr}\left(x>\hat{X}\right)=\int_{\hat{X}}^{\infty }\frac{\frac{1}{\sigma_{Knet}}{\left(\frac{W}{r}\right)}^{\frac{1}{\sigma_{Knet}}}}{x^{{\left(\frac{W}{r}\right)}^{\frac{1}{\sigma_{Knet}}+1}}}\;dx={\left(\frac{W}{r\hat{X}}\right)}^{\frac{1}{\sigma_{Knet}}}$$

The wealth possessed by residents at the quantile *q* level, where their wealth rank is in the upper quantile *q*, is denoted as *x*(*q*). According to Equation (19), it can be computed as $x\left(q\right)=w/{rq}^{\sigma_{Knet}}$. Further, the wealth of residents whose economic wealth ranking is below quantile *q* can be calculated as: $T\left(q\right)=qE\left(x|x\geq x\left(q\right)\right)=\frac{Wq^{1-\sigma_{Knet}}}{r\left(1-\sigma_{Knet}\right)}$.

To obtain the share of total national wealth held by residents above the *q* quantile, it is sufficient to divide *T*(*q*) by the total national wealth. This share represents the top percentile share:

(20) $$S\left(q\right)=\frac{T\left(q\right)}{X}=q^{1-\sigma_{Knet}}$$

When the net capital share is larger, the wealth share of residents above the *q* quantile is also larger, indicating greater wealth inequality in the economy.^2^ Additionally, using the top percentile share equation (20), one can derive the Lorenz curve of wealth in the economy and calculate the Gini coefficient (GI):

(21) $$GI=\frac{\sigma_{Knet}}{2-\sigma_{Knet}}$$

### 4.5 The influence of AI technology on wealth distribution

In the preceding exposition, Equation (16) establishes a correlation between the real interest rate (r) and artificial intelligence technology. Equation (17) demonstrates a positive association between *r* and net capital share (*σ*_*Knet*_), while Equations (20), (21) explicate the positive relationship between net capital share and wealth distribution inequality. Herein, we expound further on the direct linkage between net capital share and wealth distribution inequality.

Primarily, within the economic framework, the aggregate wealth of each resident undergoes partition into human wealth and financial wealth. Consequently, the wealth allocation belonging to a specific demographic can be dissected into financial wealth allocation and human wealth allocation. Hence, the wealth distribution of residents surpassing the *q*th percentile (S (*q*)) can be disentangled as follows:

(22) $$S\left(q\right)=\sigma_{Knet}S_{k}\left(q\right)+\left(1-1-\sigma_{Knet}\right)S_{l}\left(q\right)$$

Taking the derivative of Equation (22), we get:

(23) $$dS\left(q\right)=\left[S_{k}\left(q\right)-S_{l}\left(q\right)\right]{d\sigma }_{Knet}+\sigma_{Knet}dS_{k}\left(q\right)+\left(1-\sigma_{Knet}\right){dS}_{t}\left(q\right)$$

Equation (23) demonstrates that the net capital share is influenced through two channels: the composition effect [*S*_*k*_(*q*)-*S*_*t*_(*q*)]*dσ*_*Knet*_ and the internal variation of factor share*σ*_*Knet*_d*S*_*k*_(*q*) +(1-*σ*_*Knet*_)d*S*_*t*_(*q*). Firstly, within the economy, the unequal distribution of financial wealth surpasses that of human wealth, i.e., [*S*_*K*_ (*q*)-*S*_*t*_ (*q*)] >0. The increase in net capital share impacts overall wealth distribution inequality through this composition effect, as the rise in net capital share enhances the significance of financial wealth in the total wealth distribution. Secondly, due to the assumption of uniform abilities among laborers in the model, changes in net capital share do not affect *S*_*t*_ (*q*), but they do affect the top share of financial wealth. An increase in net capital share results in an elevation of the top share of financial wealth because affluent individuals already possess more financial wealth, and an increase in net capital share allows them to accumulate financial wealth at a faster rate.

The increase in *β* (*AAK*)^*ε−1*^, as indicated by equation (15), will cause the capital demand curve to shift to the right, thereby raising the real interest rate. Conversely, a decrease in *β* (*AAK*)^*ε−1*^ will lower the real interest rate. If *ε* > 1, implying total substitutability among various tasks in the economy, the impact of artificial intelligence on technological progress in any form will lead to an increase in β (*AAK*) ε^*−1*^, raising the real interest rate and widening wealth inequality in the economy. Ultimately, the development of artificial intelligence technology may result in absolute inequality in the economy.

However, empirical investigations conducted by Nordhaus [32] and Acemoglu & Restrepo [5] reveal that the substitution elasticity among tasks in practical settings is observed to be below 1. Under these conditions, if the influence of artificial intelligence on technological progress is predominantly reflected in the enhancement of capital-augmenting technology, labor-augmenting technology, and Hicks-neutral technology, it will not exacerbate wealth inequality and may even reduce wealth inequality in the economy. Conversely, if the development of artificial intelligence is more focused on the advancement of automation levels, it will widen wealth distribution inequality.

Therefore, the impact of artificial intelligence technology on wealth inequality depends on whether it increases or decreases *β* (AAK) ε^−1^. If artificial intelligence technology increases β (AAK) ε^−1^, wealth distribution inequality tends to expand, and conversely, it tends to diminish.

From an intuitive standpoint, the enhancement of automation levels signifies an expanded domain in the capital production tasks, wherein labor is confined to a more delimited range of productive functions. The inter-task complementarity, coupled with heightened automation, tends to result in a relative surplus of labor, consequently engendering a reduction in the labor share. Technologies oriented towards augmenting capital serve to optimize capital efficiency, culminating in an augmentation of the effective capital stock and a relative surplus of capital, thereby leading to a concomitant diminution in the relative share of capital.

Hicks-neutral technology elevates the overall economic output, contributing to an elevated per capita capital stock during steady-state conditions. This amplification in capital surplus induces a subsequent decrement in the capital share. Notably, while labor-augmenting technologies exhibit the capacity to enhance the effective labor input, the concomitant escalation in capital accumulation mitigates the impact of such technologies on factor shares. The relative proportions of capital and labor shares function as key indicators elucidating the composition of total wealth, thereby illuminating the relative significance of human capital vis-à-vis financial wealth.

Residents with a brief tenure in the economic landscape typically possess a diminished quantum of financial wealth, in contrast to their counterparts with an extended residence period, who accumulate comparatively greater financial assets. The amplification in the capital share accentuates the centrality of financial wealth, thereby contributing to an exacerbation of wealth inequality.

### 4.6 Dynamic transfer

When the economy deviates from equilibrium, factor prices undergo changes over time. Consequently, the dynamic programming problem faced by residents will also undergo alterations. At this juncture, the dynamic programming problem for residents can be expressed through the Hamilton-Jacobi-Bellman (HJB) equation as:

(24) $$\rho V\left(a,t\right)=\max\;\frac{{c\left(t\right)}^{1-\theta }}{1-\theta }+\frac{\partial V\left(a,t\right)}{\partial a}\left[W\left(t\right)+r\left(t\right)a-c\left(t\right)\right]+\frac{\partial V\left(a,t\right)}{\partial a}$$

$$st:\dot{a}=W\left(t\right)+r\left(t\right)a-c\left(t\right)$$

where c(t) represents the consumption level of an individual resident at time t. The marginal propensity to consume, denoted by μ(t), is defined as the ratio of consumption to wealth, i.e., *c*(*t*) = *μ*(*t*)*x*(*t*). By solving the Hamilton-Jacobi-Bellman (HJB) equation and examining its necessary conditions, we obtain the Euler equation: $\frac{\dot{\mu }\left(t\right)}{\mu \left(t\right)}=\mu \left(t\right)-r\left(t\right)+\frac{1}{\theta }\left(r\left(t\right)-\rho \right)$. The accumulation equation of individual wealth can also be obtained: $\dot{x}\left(t\right)=\left(r\left(t\right)-\mu \left(t\right)\right)x\left(t\right)$.

The accumulation function of individual wealth yields the Kolmogorov forward equation corresponding to the density function of individual resident wealth distribution:

(25) $$\frac{\partial g\left(x,t\right)}{\partial t}=-\frac{\partial }{\partial x}\left[\left(r\left(t\right)-\mu \left(t\right)\right)xg\left(x,t\right)\right]-pg\left(x,t\right)+p\hat{\delta }\left[x-\frac{W\left(t\right)}{r\left(t\right)}\right]$$

where $\hat{\delta }\;\left(\cdot \right)$ represents the Dirac Delta function. Equations (24), (25) together constitute a coupled partial differential equation system. By solving equations (24), (25), the model provides the consumption and saving policy functions, factor prices, capital stock, economic output, and the distribution function of wealth for individual residents at each moment in time.

## 5 Numerical simulation

In this section, Chinese data are used as the calibration basis for the model. Through numerical simulations, we explore the impact of artificial intelligence technology on wealth inequality in both the long term and short term. It is important to note that the goal of the numerical simulations in this paper is not to accurately predict the future but rather to investigate the scope and direction of the economic impact of artificial intelligence technology. This section is divided into two parts:

The first part involves a sensitivity analysis of the long-term steady state with respect to technological parameters, revealing the influence of artificial intelligence on wealth distribution in the long-term steady state.

The second part examines the dynamic transition paths of the model after being subjected to shocks from changes in technological parameters, illustrating how the application of artificial intelligence technology affects the economy in both the short term and long term over time. The year 2010 is chosen as the initial point for the model's transfer dynamics and sensitivity analysis. The model requires the calibration of five parameters and four baseline technological levels: *θ*, *p*, *δ*, *ϑ*, *ε,* and *β*_*0*_, *A*_*0*_, *A*_*K0*_, *A*_*L0*_*,* respectively.

*θ* represents the intertemporal elasticity of substitution, and the literature commonly adopts the standard parameter value *θ* = 3. *p* represents the mortality rate, and the average value for China from 1995 to 2019 is used, set at 0.007. The value of the capital depreciation rate *δ* is referenced from existing Chinese literature estimating the capital depreciation rate for China, and it is set at *δ* = 0.1 [17,33,34].

Given the relation *ρ = ϑ* + *p*, the calibration of *ρ* facilitates the derivation of the calibrated value for *ϑ*. The calibration of parameter *ρ* employs Equation (16) as the target, i.e., *ρ = r*-*θpσ*_*Knet*_. Capital share is calculated using income approach GDP data from the National Bureau of Statistics, yielding *σ*_*K*_ = 0.49. Subsequently, the net capital share is derived as *σ*_*Knet*_ = 0.34. The actual interest rate r is equal to the average interbank lending rate provided by the People's Bank of China from 2006 to 2015, leading to *ϑ* = 0.021.

*ε* represents the elasticity of substitution between tasks, and past literature typically uses data from O∗NET for calibration [35]. Since China lacks similar data, we adopt the estimate from Acemoglu & Restrepo [5] with *ε* = 0.8 for calibration.

Based on the estimates by Acemoglu & Restrepo [5], the average cost savings of tasks after automation, in a broad sense, are approximately 30 % compared to before automation. Therefore, it can be inferred that *WA*_*K0*_/*RA*_*L0*_ = 1.3. As Hicks-neutral technology can be combined with labor-augmenting and capital-augmenting technologies, the baseline value for Hicks-neutral technology is set as *A*_*0*_ = 1. Calibration of the baseline technological levels is performed based on the demand functions for capital and labor in Equations (8), (9) as calibration targets.

The value of labor wages is determined based on the average per capita wage of urban residents calculated from the income approach GDP data of the National Bureau of Statistics from 2006 to 2015 in China. The real interest rate remains the same as previously, utilizing the interbank lending rate from 2006 to 2015 in China. The capital-output ratio is obtained from the averages estimated by Zhang et al. [17], Wang &Bai [33], and Chen [34], resulting in *β*_*0*_ = 0.7, *A*_*K0*_ = 0.14, and *A*_*L0*_ = 0.47.

### 5.1 Sensitivity analysis of steady state

Utilizing the specified calibrated parameters, compute the model's equilibrium values for interest rates, wages, output, and other variables. Subsequently, evaluate the disparity in wealth distribution at the equilibrium state. The subsequent analysis will examine the repercussions of variations in automation levels, Hicks-neutral technology, capital-augmenting technology, and labor-augmenting technology on output, interest rates, wealth distribution inequality, and the well-being of distinct income brackets. Throughout this analysis, other technologies remain constant. The Gini coefficient is employed to gauge wealth distribution inequality, while value functions measure changes in welfare for different income strata.^3^

**Fig. 4:**
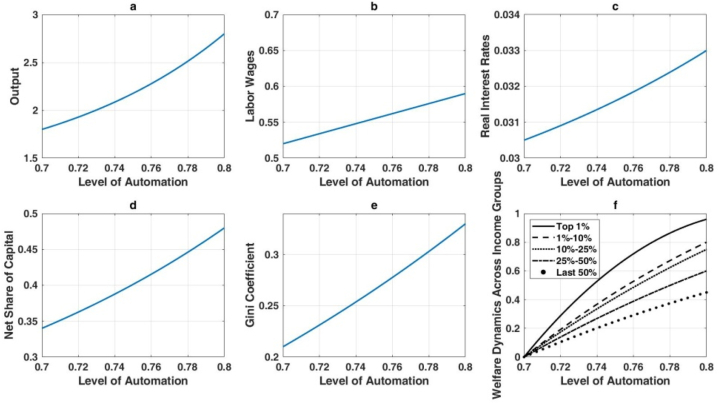
Long-term economic implications of elevated automation levels.

As proposed in Hypothesis 1, advancements in AI-driven automation exacerbate wealth inequality, particularly between capital and labor incomes. This relationship is illustrated in Fig. 4. Increasing automation by 0.1 units relative to the baseline results in a 58 % rise in steady-state output, as depicted in Fig. 4(a), which highlights the substantial productivity gains from automation. The resulting economic expansion also drives up wages, as shown in Fig. 4(b), reflecting the higher overall productivity. Simultaneously, Fig. 4(c) demonstrates a rise in real interest rates, driven by intensified capital demand. Fig. 4(d) underscores the increasing net capital share, highlighting how automation disproportionately enhances capital income and skews the income distribution. Moreover, Fig. 4(e) reports a 32 % increase in the Gini coefficient, signifying widening wealth disparities. While all income groups experience welfare gains (Fig. 4(f)), the benefits are uneven, with wealthier individuals realizing more significant absolute gains, thereby intensifying inequality.

**Fig. 5:**
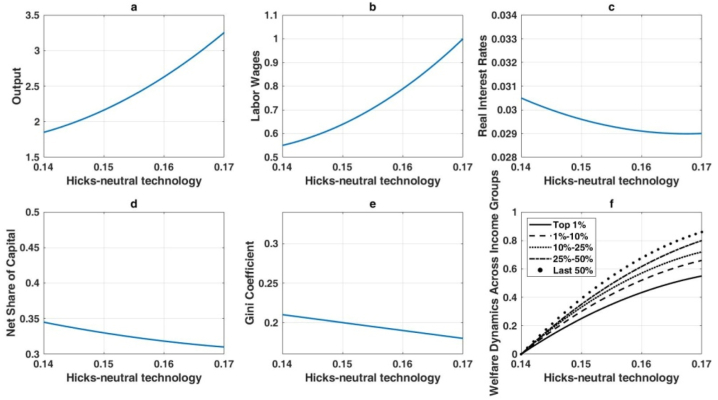
The long-term impact of hicks-neutral technological advancements on the economy.

Fig. 5 illustrates the effects of Hicks-neutral technological progress, which improves both labor and capital productivity simultaneously. This form of technological advancement enhances output, wages, and real interest rates while reducing wealth inequality. Fig. 5(a) shows a pronounced increase in output, driven by balanced productivity gains. Fig. 5(b) highlights robust wage growth, as labor productivity rises in tandem with capital productivity. Although real interest rates also increase, as shown in Fig. 5(c), the changes are less dramatic than under automation, reflecting a more balanced economic environment. Fig. 5(d) reveals a relatively lower net capital share, indicating a proportionate rise in labor income. Consequently, the Gini coefficient decreases (Fig. 5(e)), demonstrating that balanced technological improvements help narrow wealth disparities. Furthermore, Fig. 5(f) shows that lower-income groups benefit significantly due to their reliance on wages, resulting in more equitable welfare gains.

**Fig. 6:**
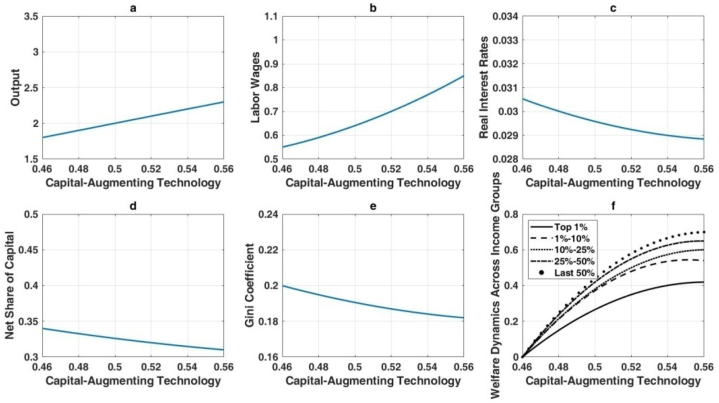
Long-term economic impact of capital-augmenting technology enhancement.

Hypothesis 2 posits that capital-augmenting technological progress increases output, wages, and real interest rates while moderating wealth inequality, mirroring some effects of Hicks-neutral advancements. These outcomes are detailed in Fig. 6. Capital productivity enhancements drive a 47 % increase in output for a 20 % rise in the capital-augmenting component, as shown in Fig. 6(a), though this is less pronounced than the 82 % gain observed with Hicks-neutral technology. Wages also increase (Fig. 6(b)), but with a more pronounced skew toward capital returns. Fig. 6(c) highlights elevated real interest rates, consistent with higher capital demand. The expanding net capital share in Fig. 6(d) reflects the disproportionate efficiency gains for capital. Nevertheless, Fig. 6(e) shows a reduction in overall wealth inequality, as wage earners benefit from productivity gains, albeit with a structural advantage for capital owners. Welfare gains are proportionally larger for lower-income groups (Fig. 6(f)), but wealthier individuals still capture substantial absolute improvements.

**Fig. 7:**
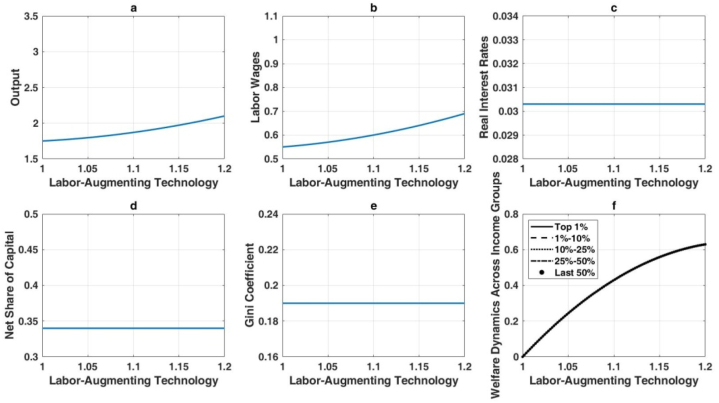
Long-term impact of labor-augmenting technological advancements on the economy.

The effects of labor-augmenting technological progress are depicted in Fig. 7. As labor efficiency improves, output experiences significant growth (Fig. 7(a)). Workers benefit directly from these improvements, as indicated by the marked wage increases in Fig. 7(b). In contrast, Fig. 7(c) shows that real interest rates remain stable, reflecting a steady long-term capital-to-labor ratio. Fig. 7(d) confirms that the net capital share remains largely unchanged, as labor-augmenting technology does not shift factor shares in steady state. Wealth inequality, as measured by the Gini coefficient, also remains stable (Fig. 7(e)), given the balanced nature of labor-augmenting improvements. Finally, Fig. 7(f) demonstrates uniformly higher welfare across all income groups, reflecting equitable gains that do not exacerbate distributional disparities.

Overall, these findings reveal that automation-driven AI strongly boosts output but intensifies wealth inequality, while Hicks-neutral and capital-augmenting technologies foster broad-based growth and mitigate wealth disparities. Labor-augmenting progress raises wages and output without affecting wealth distribution. The analysis underscores the nuanced role that different types of AI-induced technological change play in shaping long-term economic outcomes and income distribution.

The enduring outcomes of numerical simulations in this study substantiate the earlier theoretical analysis, revealing that the impact of AI technology on wealth distribution varies significantly based on the type of AI application. When AI advancements are primarily in automation, there is a marked tendency for wealth inequality to worsen. This is exemplified by the rise of automated manufacturing and logistics systems, where initial job losses among low-skilled workers lead to greater income disparities. However, if these workers receive effective retraining for new roles created by AI, such as in robotics maintenance or system management, the long-term inequality could be mitigated.

Conversely, AI applications that enhance capital or are Hicks-neutral demonstrate a tendency to improve wealth inequality. For instance, AI-driven innovations in financial services, like robot-advisors, democratize investment opportunities, enabling broader access to financial markets and potential wealth accumulation for a more diverse population. Similarly, AI in agricultural technologies can boost productivity and reduce costs, benefiting small farmers and leading to a more equitable distribution of income. Interestingly, labor-enhancing AI technology, which augments human capabilities rather than replacing them, shows negligible impact on wealth distribution. An example of this is AI-powered tools in healthcare that assist doctors in diagnostics without eliminating the need for medical professionals. Such technologies enhance the quality of care without significantly altering the income distribution among healthcare providers.

This study expands upon the work of Moll et al. [36] by examining a broader array of AI technologies. While our findings corroborate Moll et al. [36] in that automation tends to increase wealth inequality, our analysis also includes capital-enhancing and Hicks-neutral technologies, which were not explored in their study. For instance, AI applications in renewable energy (Hicks-neutral) contribute to long-term economic benefits by reducing energy costs and promoting environmental sustainability, which can have a more balanced impact on wealth distribution. To illustrate, consider the implementation of AI in smart grid technology. Initially, the benefits might be concentrated among large utility companies, potentially increasing inequality. However, as the technology matures and becomes accessible to smaller providers and consumers, the cost savings and efficiency gains can lead to lower energy prices and more equitable wealth distribution across different socio-economic groups. Moreover, AI technologies that enhance capital, such as automated investment platforms, allow individuals with smaller capital to participate in wealth-generating activities previously accessible only to the wealthy. This can reduce barriers to entry and promote a more inclusive financial ecosystem, ultimately contributing to a reduction in wealth inequality.

In conclusion, this study's empirical results provide a nuanced understanding of how different AI technologies affect wealth distribution. Policymakers should consider these distinctions when designing strategies to integrate AI into the economy, ensuring that the benefits are widely distributed. Initiatives such as targeted retraining programs, support for small businesses to adopt AI, and policies promoting equitable access to AI advancements can help mitigate short-term inequalities and foster long-term inclusive growth.

### 5.2 Transfer dynamics

The previous section provided an analysis of the long-term impact of AI technology on the economy. This section continues to examine the effects of changes in AI technology on output and wealth distribution when the economy deviates from its steady state. Based on the theoretical analysis presented earlier, the impact of AI technology on wealth distribution in the economy is uncertain. The specific direction depends on the extent of AI enhancement for different types of technologies.

Hypothesis 3 posits that AI adoption increases regional wealth inequality. The simulation results illustrate that the introduction of automation and capital-augmenting technologies in advanced regions leads to higher capital returns, contributing to regional disparities in wealth. Therefore, the analysis in this section is divided into two scenarios:Quantitatively, it depends on the impact of AI on the size of the parameter combination β(AAK)ε-1. Therefore, the analysis in this section is divided into two scenarios:

Scenario One, where AI technology increases β(AAK)ε-1 while keeping other technological levels constant, with an increase in automation level by 5 %.

Scenario Two, where AI technology increases β(AAK)ε-1 while keeping other technological levels constant, with a 2 % increase in automation technology and a 2.5 % increase in capital-enhancing technology.

These two scenarios are chosen because they represent plausible variations in AI technology's impact on the economy, allowing us to isolate the effects of automation and capital enhancement while holding other variables constant [4,5]. This approach enables a clearer understanding of how specific technological advancements influence economic outcomes [8,16].

**Fig. 8:**
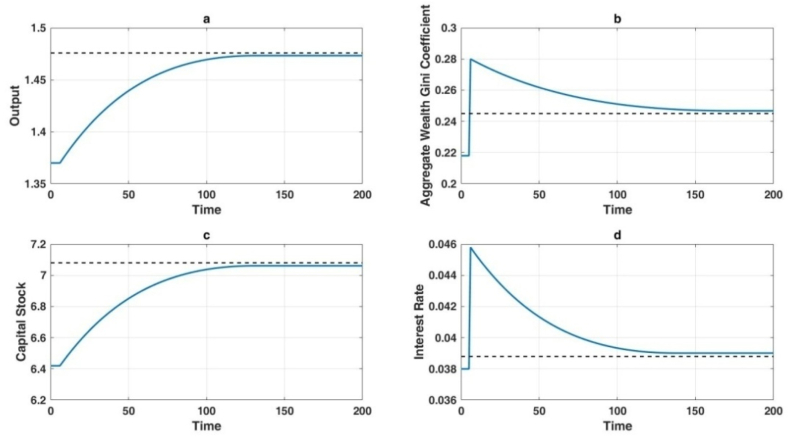
Long-term economic impact of labor-augmenting technological advancements.

**Fig. 9:**
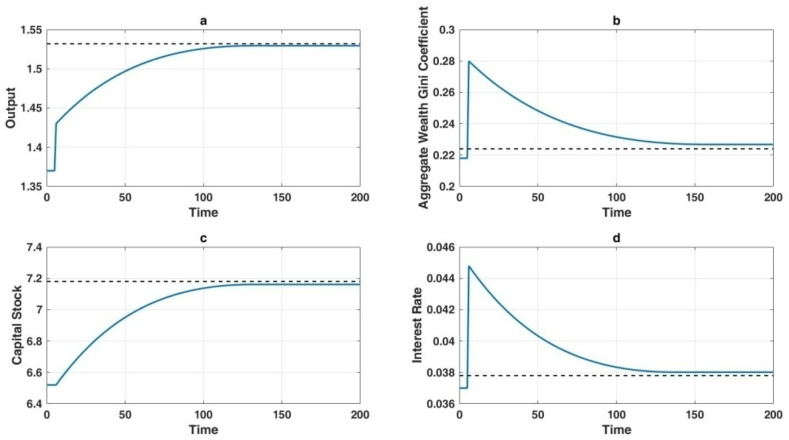
Simultaneous short-term and long-term impact of automation levels and capital-augmenting technology on the economy.

Fig. 8, Fig. 9 correspond to the transfer dynamics of the technological shocks in these two scenarios, respectively. From both scenarios, it can be observed that the impact of different technological shocks on output and capital stock is consistently positive, both in the long term and the short term. In contrast to output and capital stock, the impact of artificial intelligence technology on wealth distribution inequality and interest rates differs in the long term and the short term.

Although the long-term impact of technology on wealth distribution depends on the variation of the technological parameter combination β(AAK)ε-1, in the short term, both interest rates and the Gini coefficient exhibit overshooting phenomena. This is because technological improvements lead to an increase in capital stock in the long-term steady state. However, in the short term, capital stock cannot adjust immediately, making capital a relatively scarce factor and causing interest rates to jump upward in the short term.

The transient surge in interest rates, while not impacting the allocation of financial resources, precipitates a reduction in the ratio of human wealth to aggregate wealth for all individuals. Consequently, the disproportionate dispersion of financial assets becomes a more significant contributor to overall wealth inequality. In the short term, the overcorrection of interest rates corresponds to a parallel overcorrection in the disparity of total wealth distribution.

In Scenario One, illustrated in Fig. 8(b), the escalation in levels of automation not only results in a near-term augmentation of wealth inequality but also perpetuates an enduring increase in wealth distribution disparity. Contrastingly, upon examining Fig. 8(b) in Scenario Two, it becomes evident that while technological advancements initially trigger an overcorrection of interest rates and an expansion of wealth distribution inequality, in the long term, there is a tendency for wealth distribution inequality to stabilize. Hence, relative to the relatively pessimistic findings of Moll et al. [36], this paper presents a relatively sanguine prospect. If the outcomes of the advancements in artificial intelligence technology can be implemented in a certain manner within economic production, it may be plausible for the economy to endure greater wealth inequality in the short term, while averting the enduring impact of artificial intelligence technology on wealth inequality in the long term.

Scenario Two offers a mechanism to maintain constant wealth inequality by concurrently elevating levels of automation and capital-enhancing technology, thereby preserving the overall parameter combination β(AAK)ε-1. Consequently, the long-term impact of artificial intelligence on wealth inequality remains negligible. As illustrated in Fig. 8, labor-augmenting technology contributes to output gains while having limited effects on long-run wealth distribution.

Fig. 8 provides insights into the dynamic responses of key economic variables under enhanced labor productivity. Fig. 8(a) illustrates a steady rise in output over time, converging to a higher steady state relative to the baseline (dashed line). Fig. 8(b) shows the Gini coefficient for total wealth, which remains relatively stable, indicating minimal distributional shifts due to uniform improvements in labor productivity. Fig. 8(c) depicts a gradual upward adjustment in the capital stock, driven by indirect effects of higher labor productivity on investment. Meanwhile, Fig. 8(d) highlights negligible changes in real interest rates at the new steady state, as labor augmentation does not significantly affect the long-run capital–labor ratio.

Fig. 9 explores the scenario where automation and capital-augmenting technology advance simultaneously. Fig. 9(a) demonstrates substantial long-term gains in aggregate output, reflecting the combined effects of increased mechanization and improved capital efficiency. Fig. 9(b) captures a short-term rise in the Gini coefficient, indicative of initial job displacement and capital-share expansion, followed by a gradual decline as workforce adaptation and capital diffusion mitigate inequality. Fig. 9(c) reveals a pronounced surge in the capital stock, driven by the immediate demand for physical capital and the enhanced returns from capital-augmenting innovation. Finally, Fig. 9(d) shows an initial spike in real interest rates, which subsequently moderate as the expanded capital base alleviates upward pressure on capital demand.

Empirically, one observes parallel phenomena in real-world settings. For example, automating manufacturing processes can displace lower-skilled labor, thereby triggering a short-lived rise in inequality. Over time, however, reskilling and reallocation allow these workers to occupy AI-supported roles such as maintenance, oversight, and more specialized positions, fostering widespread productivity growth. This trajectory aligns with the temporary increase in the Gini coefficient in Fig. 8(b) or Fig. 9(b) and its eventual normalization. Likewise, the early introduction of automated trading systems in financial markets initially advantaged a small group of highly skilled traders, widening wealth disparities. As the technology diffused and became more accessible, aggregate gains accrued through greater market efficiency and liquidity. A parallel case emerges in healthcare, where cutting-edge AI diagnostic tools initially benefited higher-income individuals yet ultimately conferred significant improvements in treatment outcomes once they proliferated.

The numerical simulations underscore the idea that while AI-driven technological progress can temporarily aggravate wealth inequality, it also promotes substantial, sustained improvements in economic output—as shown by the upward shift in Fig. 9(a). Over the long term, this can represent a Pareto improvement, provided that short-run disruptions are addressed through effective policies. The enduring consequences for the wealth distribution depend on the specific mixture of automation, labor-augmenting innovation, and capital-augmenting technology that unfolds. Consequently, policymakers seeking to harness the long-term econ omic potential of AI must prioritize inclusive strategies, such as worker retraining initiatives, support for equitable access to emerging technologies, and frameworks that facilitate broad-based participation in AI-driven growth. By doing so, societies can realize AI's aggregate productivity gains—depicted in the upward trajectories of output in Fig. 8, Fig. 9—while mitigating the transient surges in inequality evidenced by Fig. 8, Fig. 9.

## 6 Conclusions, implications and future research directions

### 6.1 Conclusions

This article undertakes an economic analysis of the ramifications arising from the integration of AI technology into economic production. The consequences are examined in terms of fostering automation, enhancing labor efficiency, advancing capital-centric technologies, and driving Hicks-neutral technological progress. Using a heterogeneous agent dynamic general equilibrium model, two key conclusions are drawn.

Firstly, the adoption of AI technology consistently leads to a relative scarcity of capital, which results in an increase in the return on capital and the capital share. This, in turn, exacerbates wealth inequality. This finding aligns with recent studies, such as Acemoglu et al. [35], who highlighted that increased automation tends to worsen wealth inequality by disproportionately benefiting capital over labor.

Secondly, the overall effects of AI on wealth distribution depend on the relative advancement of four categories of technological progress. While heightened automation worsens wealth inequality, capital-enhancing technologies and Hicks-neutral technological progress help mitigate these disparities. This nuanced perspective deepens the conclusions of recent research by Aghion et al. [15], which suggested that different types of technological progress have varied impacts on economic inequality. Specifically, Aghion et al. noted that capital-enhancing technologies could reduce inequality—a finding that our study quantitatively supports and expands upon by also considering Hicks-neutral progress.

Further numerical analysis shows that policies fostering AI technological progress generally contribute to output growth and improve welfare. AI is poised to become a new catalyst for economic expansion in China, injecting vitality into the economy. However, amidst the growth spurred by AI, it is essential to address its impact on wealth distribution, particularly in the short term. Our findings are consistent with Zhang et al. [17], who also noted AI's significant potential to drive economic growth while emphasizing the need for policies to address the resulting inequality.

Nonetheless, the short-term redistributive effects of AI lead to a widening of wealth inequality. This observation aligns with concerns raised by Van Reenen (2021)32, who emphasized that capital-biased technological changes exacerbate inequality. In contrast to the short-term effects, the long-term redistributive impacts of AI seem more measured and may even help alleviate wealth inequality under certain conditions. This suggests that while the immediate impacts may pose challenges, strategic policy interventions can help mitigate these effects over time, as discussed by Acemoglu and Johnson [35].

Therefore, in the short term, the dual objectives of reducing wealth inequality and capitalizing on the benefits of AI-driven growth can only be achieved through secondary distribution mechanisms. Taking a long-term view, the factors driving wealth inequality due to AI adoption largely stem from an increase in automation levels. However, advancements in capital-enhancing and Hicks-neutral technologies can help alleviate the extent of inequality. Thus, policy choices that balance growth incentives with wealth distribution objectives should prioritize fostering progress in capital-enhancing and Hicks-neutral technologies.

In conclusion, this study offers a nuanced understanding of AI's economic impacts, highlighting the differentiated effects of various types of technological progress on wealth distribution. By integrating comprehensive modeling with empirical analysis, this research provides actionable insights that contribute to both theoretical discourse and practical policymaking, expanding upon and aligning with existing literature.

### 6.2 Implications

#### 6.2.1 Implication for theory

This study makes several original theoretical contributions that address significant research gaps in understanding the economic impact of AI.

First, by extending the analysis to include property income and wealth inequality, the study provides a more comprehensive understanding of how AI affects overall wealth distribution. This approach broadens the traditional focus of general equilibrium modeling, which typically centers on income measures, by offering a more detailed analysis of wealth disparities. As a result, it provides a richer theoretical perspective on the broader implications of AI for economic inequality.

Second, the study employs a continuous-time heterogeneous agent dynamic general equilibrium model with a task-based production perspective. This innovative methodology allows for a nuanced examination of AI's impact across various economic sectors, significantly enhancing the theoretical understanding of how technological advances interact with different production processes. The model incorporates multiple forms of technological progress—automation, capital-augmenting, labor-augmenting, and Hicks-neutral—offering a multifaceted view of AI's economic implications. This methodological choice is crucial as it integrates both theoretical and empirical analyses, strengthening the robustness of the findings and providing a comprehensive perspective on the economic effects of AI.

Third, calibrating the model using Chinese data provides valuable insights into how AI influences wealth inequality in rapidly developing economies. The contextual specificity of the Chinese economy enriches the theoretical framework by incorporating empirical evidence from a significant and unique economic setting. This allows the study's findings to be not only applicable to China but also relevant to similar emerging markets, thus broadening the generalizability of the theoretical insights.

Finally, the study's actionable policy interventions bridge the gap between theory and practice. It proposes measures to mitigate the rising wealth inequality resulting from AI advancements, offering concrete solutions to real-world economic challenges. This focus on both theoretical analysis and practical policy underscores the importance of research that addresses the multifaceted impact of AI on wealth distribution.

In sum, the contributions of this study significantly advance the theoretical understanding of the economic impact of AI. By integrating comprehensive wealth measures into the analysis, employing innovative modeling techniques that account for various forms of technological progress, using context-specific data to provide nuanced insights into rapidly developing economies, and combining theoretical insights with practical policy recommendations, the study provides a robust foundation for future research in this critical area.

#### 6.2.2 Implication for practice

This study offers valuable insights for researchers, practitioners, and policymakers, each of whom can apply these findings to their respective fields.

For researchers, this study provides a novel framework for analyzing the economic impact of AI, particularly on wealth distribution. The continuous-time heterogeneous agent dynamic general equilibrium model, coupled with a task-based production perspective, opens new avenues for examining how AI affects different sectors. Researchers can leverage this model to explore the intricate dynamics of AI's role in enhancing capital and labor productivity, thus enriching the academic discourse on technological progress and economic inequality.

For practitioners, the study emphasizes the importance of focusing on AI innovations that enhance capital productivity. Professionals in key sectors such as manufacturing, energy, and logistics can use these findings to guide their investment and development strategies. By prioritizing AI technologies that improve efficiency and output, practitioners can achieve significant productivity gains. Moreover, fostering collaborations between state-owned enterprises (SOEs) and private companies can facilitate the rapid deployment of AI solutions, leveraging the strengths of both sectors for optimal results.

For policymakers, the study highlights the need for targeted interventions to address the potential exacerbation of wealth inequality resulting from AI advancements. Policies should incentivize capital-intensive technological developments and promote Hicks-neutral technological progress to ensure balanced economic growth. Implementing redistributive measures, such as additional taxes on capital income and investments in education and skill development, can help mitigate short-term inequalities. Additionally, directing AI development and investments toward underdeveloped regions can address regional disparities, fostering inclusive growth through initiatives such as special economic zones and innovation hubs.

In summary, this study provides actionable insights and recommendations that can guide researchers, practitioners, and policymakers in harnessing the potential of AI technologies while addressing the challenges of wealth inequality and economic disparity. By integrating these findings into their respective domains, stakeholders can contribute to a more equitable and productive future shaped by AI-driven innovations.

### 6.3 Limitations of the study and future research directions

While this study provides important insights into the economic impacts of AI, it has several limitations that future research should address.

Firstly, the study does not capture the temporal evolution of AI technology. The current model provides a static view, which may overlook how AI advancements progress over time and their varying impacts. Future research should employ dynamic models capable of tracking the development and maturation of AI technologies. This would offer a more comprehensive understanding of how AI's influence on economic output and wealth distribution evolves.

Secondly, the study does not consider the potential for AI to cause unemployment. Increased automation could lead to job losses, significantly affecting income inequality. By focusing solely on the productivity effects of AI, the model may underestimate its broader economic consequences. Future research should integrate unemployment mechanisms to provide a more holistic analysis of AI's impact on wealth inequality.

Thirdly, while this study examines the immediate impacts of different types of technological progress on wealth distribution, it does not delve into critical aspects of AI, such as its potential to create new industries or transform existing ones. Future research should adopt a more comprehensive approach to understanding these broader ramifications of AI, offering a nuanced perspective on how AI-driven innovations might reshape economic landscapes and long-term wealth distribution.

Additionally, while the study's findings are directly applicable to policymaking, empirical validation of the proposed policy interventions in real-world settings is needed. Future studies could conduct case studies or pilot programs to test the effectiveness of these policies in mitigating wealth inequality and promoting equitable economic growth. Such empirical validation will enable policymakers to design and implement strategies based on practical evidence.

Furthermore, this study uses data from China, which is valuable for understanding AI's impact in a rapidly developing economy. However, the findings may not be fully generalizable to other contexts. Future research should apply similar models in different countries and economic environments to test the robustness of the results. Comparative studies across regions will help better understand how AI affects wealth inequality in diverse settings and provide insights into region-specific policy interventions.

In conclusion, addressing these limitations in future research will enhance the understanding of AI's economic impacts and provide more robust, actionable insights for policymakers, practitioners, and researchers. By integrating dynamic, comprehensive, and empirically validated approaches, future studies can build upon the foundations laid by this research to develop a deeper, more holistic understanding of AI's role in shaping economic outcomes. This will ultimately help in designing effective strategies to harness the benefits of AI while mitigating its potential downsides.

## CRediT authorship contribution statement

**Fang Liu:** Writing – original draft, Formal analysis, Conceptualization. **Chen Liang:** Investigation, Data curation.

## Data availability statement

The data that support the findings of this study are available on request from the corresponding author. The data are not publicly available due to privacy or ethical restrictions.

## Ethics declaration

Review and/or approval by an ethics committee as well as informed consent was not required for this study because this article did not involve any direct experimentation/studies on living beings.

## Declaration of competing interest

The authors declare that they have no known competing financial interests or personal relationships that could have appeared to influence the work reported in this paper.

## Footnotes

1 In this context, the paper does not consider how the four technical parameters *β*, *A*, *A*_*K*_, and *A*_*L*_ change over time. Uzawa (1965) demonstrated that in growth models that utilize labor and accumulable capital for production, the economy exhibits a balanced growth path only when technological progress is purely labor-augmenting.

2 For example, when q is set at 20 %, *S* (0.2) represents the wealth owned by the top 20 % of the affluent population. With a net capital share of 50 %, the corresponding wealth share for these affluent individuals is 44.72 %. However, with a net capital share of 65 %, the wealth share held by the top 20 % of the affluent population increases to 59.93 %.

3 In a more specific formulation, the value function for the resident group is computed by setting V(*x*(*q*);*β*,*A*,*A*_*K*_,*A*_*L*_) = *V*((1 + *γ*)*x*(*q*);*β*_0_,*A*_0_,*A*_*K0*_,*A*_*L0*_), where *β*_*0*_*,A*_*0*_*,A*_*K0*_*,A*_*L0*_represent the baseline values for the four types of technology, and *γ* denotes the level of welfare improvement resulting from technological advancement. In other words, *γ* quantifies the extent to which technological improvement contributes to wealth enhancement when technology remains constant.
